# Lung cancer in patients with and without rheumatoid arthritis: A propensity score‐matched survival analysis cohort study

**DOI:** 10.1111/1759-7714.13388

**Published:** 2020-03-27

**Authors:** Lei Zhang, Qiliang Zhao, Fan Yuan, Min Liu

**Affiliations:** ^1^ First Teaching Hospital of Tianjin University of Traditional Chinese Medicine Tianjin China; ^2^ Tianjin University of Traditional Chinese Medicine Tianjin China

**Keywords:** Interstitial lung disease, lung cancer, qi deficiency, rheumatoid arthritis, survival analysis

## Abstract

**Background:**

Connective tissue disease increases the risk of lung cancer, but whether rheumatoid arthritis (RA) has an effect on the overall survival (OS) rate in this population has not been well studied.

**Methods:**

Patients diagnosed with lung cancer between January 2015 and December 2017 were enrolled in this retrospective analysis. Propensity score matching was performed to balance the baseline of the two groups, whereas the differences between patients with and without RA were compared using survival analysis. Further, the effects of interstitial lung disease (ILD) and qi deficiency on survival in cases of RA with lung cancer were examined. Cox regression analysis was applied to predict the factors that influenced the survival of lung cancer to one year.

**Results:**

Overall, 154 lung cancer patients, including 136 (88.3%) without RA and 18 (11.7%) with RA, were included. Two comparison cohorts were matched by 1:2 propensity score matching, which yielded 18 lung cancer patients with RA and 36 lung cancer patients without RA. Ultimately, the survival prognosis of lung cancer and RA was worse than that without RA, that of patients with ILD with RA and lung cancer was worse than that among those without RA, and that of patients with qi deficiency with RA and lung cancer was worse than that among those without RA.

**Conclusions:**

The survival prognosis of lung cancer patients with RA is worse than that of those without RA. ILD and qi deficiency promote reduced survival when found in conjunction with RA in patients with lung cancer.

## Introduction

Connective tissue disease (CTD) with lung cancer is a special type of lung cancer. A historical cohort study revealed that CTD increased patients' risk of lung cancer but had no significant effect on the overall survival.^1^ Available data suggest that dermatomyositis (DM), rheumatoid arthritis (RA), and systemic sclerosis (SSc)^2^, ^3^, ^4^ increase the risk of lung cancer, but no literature report on whether or not RA has an effect on the survival rate in lung cancer exists at this time. Further, ILD is a common pulmonary complication of RA and can reduce the overall survival of patients with RA, but whether ILD combined with RA would affect the overall survival of lung cancer patients has not been studied. Traditional Chinese medicine (TCM) considers RA as a toxin, but, as the disease progresses, RA will present the clinical manifestations of qi deficiency, which represents the shortened survival period of patients. We conducted a retrospective study to investigate the effects of RA with or without ILD and qi deficiency symptoms on lung cancer survival. Importantly, the life expectancy of lung cancer patients is known to be affected by various confounding factors, such as age, gender, body mass index (BMI), and pathological type, which can affect our observations. We therefore used propensity score matching (PSM) to balance the baseline of the covariates between the two groups to investigate whether RA with or without coexistence with interstitial lung disease and qi deficiency symptoms had an effect on the survival of patients with lung cancer.

## Methods

### Patient selection

**Figure 1:**
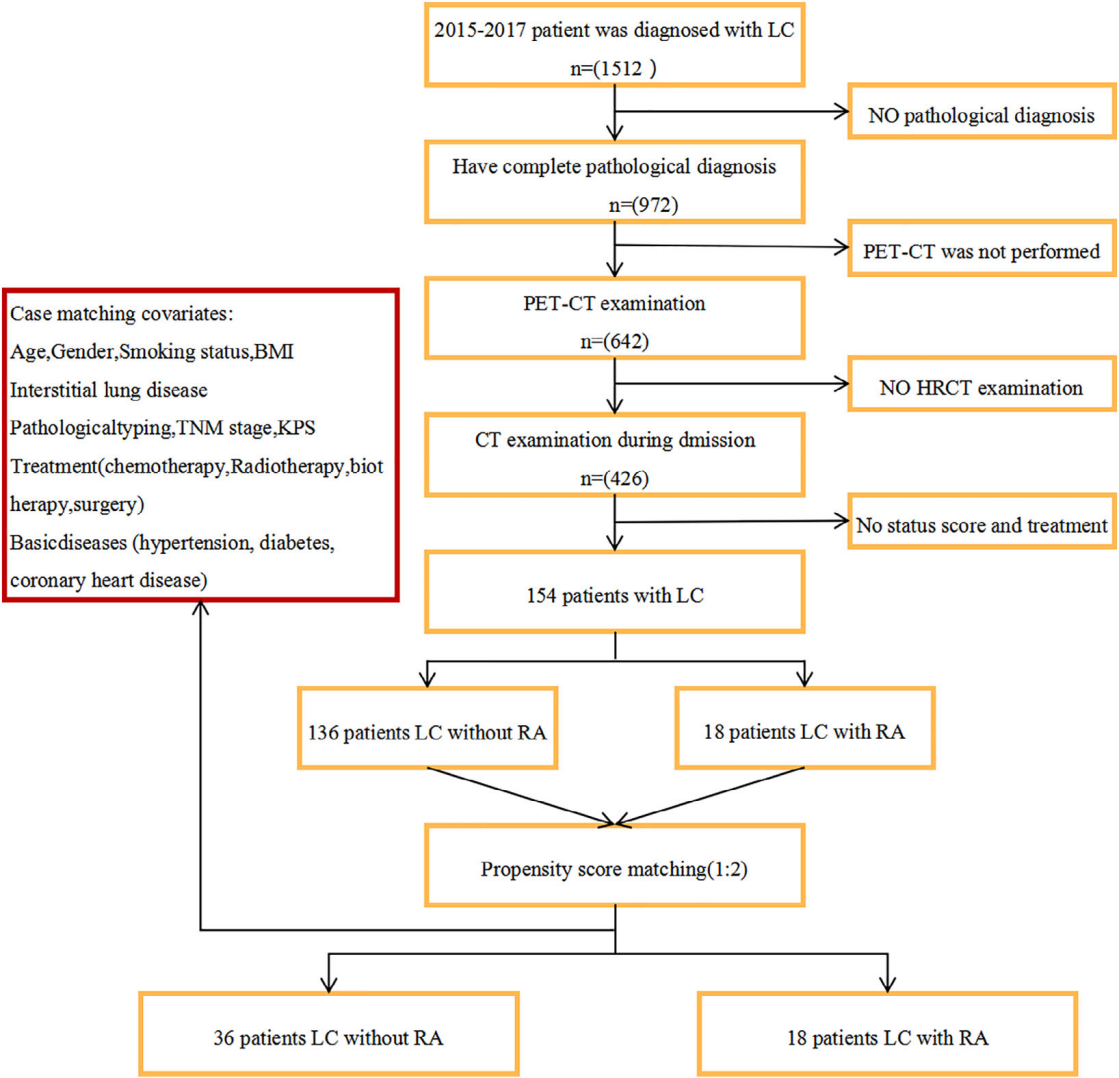
The flowchart displays the selection of lung cancer patients for analysis.

We collected relevant clinical data from the First Teaching Hospital of Tianjin University of Traditional Chinese Medicine (TJUTCM) for 154 patients who were diagnosed with lung cancer between January 2015 and December 2017 in this retrospective study. We selected 154 patients diagnosed with lung cancer for further review according to the following inclusion criteria: (i) diagnosed with lung cancer by pathological analysis; (ii) had complete clinical data (ie, positron emission tomography‐computed tomography examination and tumor, node, metastasis stage when finding tumors; HRCT examination during the first hospitalization in the TJUTCM, and Karnofsky Performance Status (KPS), and the course of treatment), and (iii) were without complications of other cancers. As a result, 18 patients diagnosed with lung cancer with coexisting RA and 36 patients with lung cancer without RA were included. We divided the patients into two groups according to whether they had RA or not, where the “case” group included those with lung cancer and coexisting RA and the “control” group included those with lung cancer without RA (Fig 1).

### Lung cancer with RA

In our study, we defined lung cancer with coexisting RA as a case of lung cancer that met the diagnostic criteria of RA and which was pathologically diagnosed. For the diagnosis of RA, a previous history of RA and adherence to the following diagnostic criteria were required: (i): Four to 10 small joints (large joints not counted) or more than 10 joints (including at least one small joint) affected; (ii) high positive RF or high positive ACPA, and (iii) abnormal CRP or abnormal ESR.^5^

### Diagnosis of ILD

In our study, the diagnosis of ILD was based on HRCT at admission. For the preliminary evaluation of HRCT in patients, the necessary characteristics were as follows: the observation of HRCT manifestations, such as honeycombing or traction bronchiectasis, which may be seen with the concurrent presence of ground‐glass opacification, fine reticulation, and so on, was required. Subsequently, we invited respiratory specialists to reevaluate HRCT findings to diagnose ILD after the initial evaluation.

### Definition of qi deficiency

In our study, patients were divided into two TCM syndrome groups according to their clinical symptoms when a tumor was found, including qi deficiency and no qi deficiency. Qi deficiency is a type of TCM syndrome, and we defined qi deficiency as meeting the major clinical symptom (ie, shortness of breath) and one or more secondary clinical symptoms (eg, frequent urination at night, sweating, fatigue, poor appetite). A case of no qi deficiency was defined as not meeting any of the clinical symptoms mentioned above.

### Evaluation index and follow‐up data collection

The primary endpoint of this study was overall survival (OS). Overall survival was measured from the day of lung cancer diagnosis until death or follow‐up to July 2019. The patients who survived over five years were considered to have survived 60 months. Patients were followed up by telephone, and we excluded the data for those whom we could not contact.

### PSM

The R version 3.6.1 software program (R Foundation for Statistical Computing, Vienna, Austria) was utilized for PSM. To adjust for the imbalance of potential confounders between the two groups, we applied PSM which used the nearest‐neighbor matching algorithm with a 1:2 matching scheme for patients with lung cancer and coexisting RA to patients with lung cancer without RA via the R package “Matching.” The propensity score was estimated, involving 14 variables: age, gender, smoking status, BMI, interstitial lung disease, pathological typing, TNM stage, KPS, treatment (eg, chemotherapy, radiotherapy, biotherapy, surgery), and basic diseases (eg, hypertension, diabetes, coronary heart disease). The 1:2 PSM yielded matched pairs of 18 subjects with lung cancer with coexisting RA and 36 patients with lung cancer without RA, with no differences in the 14 covariates. Lastly, we estimated the standardized differences in confounding factors before and after PSM using the R package “Tableone.”

## Statistical analysis

The categorical variables are expressed as frequencies and percentages, whereas the continuous variables are expressed as means ± standard deviations. We used Pearson's chi‐squared test or Fisher's exact test to analyze the differences in clinicopathological features as appropriate. The survival state was calculated by Kaplan‐Meier estimation and log‐rank test. Univariate and multivariate Cox proportional‐hazards models were used to estimate the significance level and relative risk with a 95% confidence interval (CI). *P*‐values of less than 0.05 were statistically significant. All data management and statistical analyses were conducted using the Statistical Package for the Social Sciences version 21.0 software program (IBM Corp., Armonk, NY, USA).

## Results

### Patient characteristics

Between 1 January 2015, and 31 December 2017, a total of 154 patients were included and underwent PSM, including 136 patients with lung cancer without RA and 18 patients with lung cancer and coexisting RA (Fig 1).

**Figure 2:**
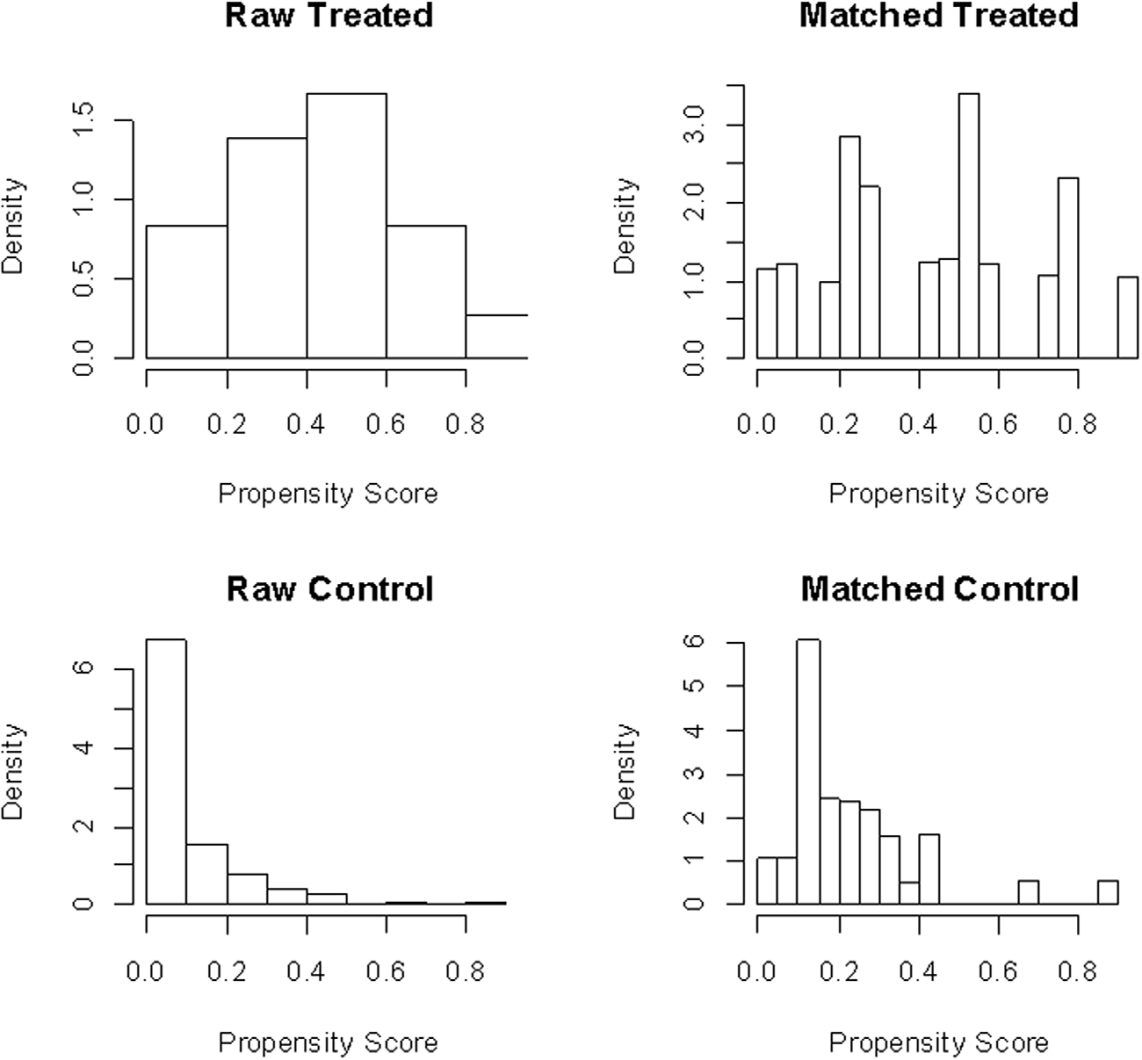
Histograms of the unmatched and matched groups. The overall balance of the six variables in the model indicates that the matching process was successful.

Upon comparing the two groups' baseline characteristics before PSM, patients with lung cancer and coexisting RA were more likely to be younger (64.35 ± 8.70 vs. 68.83 ± 5.70 years old; *P* = 0.036) and have lower KPS values (52.43 ± 17.99 vs. 65.56 ± 20.64 points; *P* = 0.005). We used the PSM method to balance the differences between the two groups. The 1:2 PSM yielded matched pairs of 18 subjects with lung cancer and coexisting RA and 36 patients with lung cancer without RA. No statistical differences were observed between the two groups as described in Table 1. In order to further study this imbalance, we analyzed the histograms of propensity score distribution before and after PSM. Figure 2a presents a histogram showing an unbalanced distribution of propensity scores in the first two groups of PSM, whereas Figure 2b presents the tendency for equilibrium of the score distribution in the histogram of the latter two groups.

**Table 1:** Demographics and pathological characteristics before and after propensity score matching

	Unmatched comparison			Matched comparison		
Characteristic	N‐RA (*n* = 136)	RA (*n* = 18)	*P*‐value	N‐RA (*n* = 36)	RA (*n* = 18)	*P*‐value
Age (years)	64.35 ± 8.70	68.83 ± 5.70	0.036	68.00 ± 6.57	68.83 ± 5.70	0.649
Gender, *n* (%)	–	–	0.170	–	–	0.921
Female	63(46.3)	12(66.7)	–	22(61.1)	12(66.7)	–
Male	73(53.7)	6(33.3)	–	14(38.9)	6(33.3)	–
BMI	21.73 ± 3.04	22.58 ± 2.52	0.254	22.65 ± 3.47	22.58 ± 2.52	0.942
Smoke = Yes, *n*(%)	80 (58.8)	13 (72.2)	0.403	24 (66.7)	13 (72.2)	0.917
ILD = Yes, *n* (%)	29 (21.3)	5 (27.8)	0.750	9 (25.0)	5 (27.8)	1.000
Pathological, *n* (%)	–	–	0.963	–	–	1.000
SC	49 (36.0)	6 (33.3)	–	12 (33.3)	6 (33.3)	–
AC	74 (54.4)	10 (55.6)	–	20 (55.6)	10 (55.6)	–
SCLC	13 (9.6)	2 (11.1)	–	4 (11.1)	2 (11.1)	–
TNM stage, *n* (%)	–	–	0.205	–	–	0.920
I	2 (1.5)	0 (0.0)	–	–	–	–
II	14 (10.3)	4 (22.2)	–	7 (19.4)	4 (22.2)	–
III	43 (31.6)	8 (44.4)	–	15 (41.7)	8 (44.4)	–
IV	77 (56.6)	6 (33.3)	–	14 (38.9)	6 (33.3)	–
KPS	52.43 ± 17.99	65.56 ± 20.64	0.005	61.94 ± 15.82	65.56 ± 20.64	0.479
CT = Yes, *n* (%)	96 (70.6)	8 (44.4)	0.050	17 (47.2)	8 (44.4)	1.000
RT = Yes, *n* (%)	62 (45.6)	7 (38.9)	0.776	14 (38.9)	7 (38.9)	1.000
BT = Yes, *n* (%)	21 (15.4)	1 (5.6)	0.443	3 (8.3)	1 (5.6)	1.000
OT = Yes, *n* (%)	28 (20.6)	3 (16.7)	0.938	6 (16.7)	3 (16.7)	1.000
HBP = Yes, *n* (%)	39 (28.7)	8 (44.4)	0.274	17 (47.2)	8 (44.4)	1.000
DM = Yes, *n* (%)	23 (16.9)	2 (11.1)	0.774	4 (11.1)	2 (11.1)	1.000
CHD = Yes, *n* (%)	30 (22.1)	7 (38.9)	0.202	8 (22.2)	7 (38.9)	0.334

AC, adenocarcinoma; BMI, body mass index; BT, biological therapy; CHD, coronary heart disease.; CT, chemotherapy; DM, diabetes mellitus; HBP, high blood pressure; ILD, interstitial lung disease; KPS, Karnofsky Performance Status; N‐RA, lung cancer without rheumatoid arthritis; OT, operative treatment; RA, lung cancer with coexisting rheumatoid arthritis; RT, radiotherapy; SC, squamous carcinoma; SCLC, small cell lung cancer; SD, standard deviation.

### Evaluation of the survival for lung cancer in patients with RA

**Figure 3:**
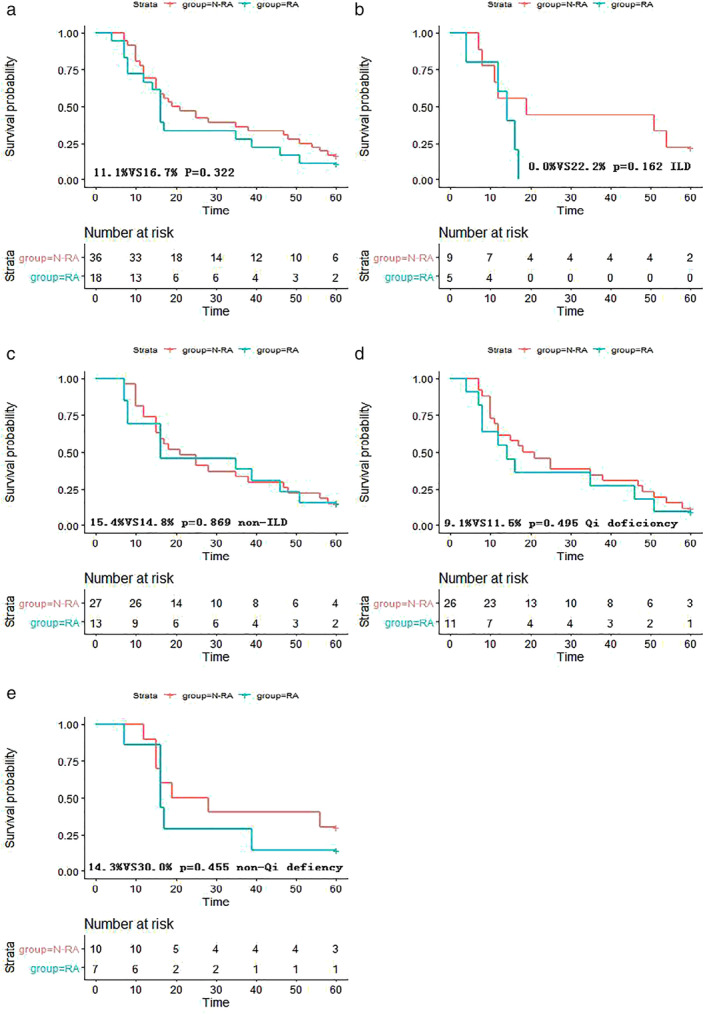
Kaplan‐Meier curves for the survival probability of patients with lung cancer and coexisting RA or without RA in the matched cohort. (**a**) Survival probability among lung cancer patients with RA and without RA. (**b**–**e**) Survival probability with different TCM syndromes, pathological types, and the presence of ILD in lung cancer patients with RA, respectively. The five‐year survival rate and the number of lung cancer patients at risk are given in each plot. 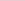 Group‐N‐RA and 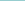 Group RA.

In this study, we compared the survival probability among lung cancer patients with or without RA. In lung cancer patients with RA (blue), the median OS was 16 months (accumulated survival rate was 11.1%), whereas in lung cancer patients without RA (red), the median OS was 19 months (accumulated survival rate was 16.7%). While this uniquely affected the OS rate of lung cancer patients according to RA status, no significant difference in survival probability was observed between the two groups (*P* = 0.322) (Fig 3(a)).

When stratifying the study population by ILD, it was found that ILD combined with RA had the greatest effect on the survival of lung cancer patients, where the median OS was 14 months (accumulated survival rate was 0%). In comparison, among lung cancer patients with ILD without RA, the median OS was 19 months (accumulated survival rate was 22.2%). However, there was no significant difference between the two RA groups in terms of OS (*P* = 0.162) (Fig 3(c)).

Considering the syndrome of TCM, in patients with qi deficiency and RA, the median OS was 14 months (accumulated survival rate was 9.1%). Conversely, among patients without RA, the median OS was 18 months (accumulated survival rate was 11.5%). Thus, there was no significant difference in the survival probability between the two groups (*P* = 0.495) (Fig 3(d)). Among patients without qi deficiency but with RA, the median OS was 16 months (accumulated survival rate was 14.3%), whereas in those without RA or qi deficiency, the median OS was 19 months (accumulated survival rate was 30%). There was also no significant difference in the survival probability between these two groups (*P* = 0.455) (Fig 3(e)).

### Prognostic risk factors for survival at 12 months in patients with lung cancer

In the univariate analysis, small cell lung cancer (SCLC) was associated with improved OS (hazard ratio (HR): 5.468, 95% CI: 1.858–16.091; *P* = 0.002) in comparison with non‐SCLC. In this study, RA, ILD, and qi deficiency affected the survival of lung cancer patients at 12 months but were not considered to be risk factors. In fact, the results of multivariate Cox proportional‐hazards regression analysis revealed that there was no main risk factor, including among smoking (HR: 9.606, 95% CI: 1.182–78.061; *P* = 0.034), not receiving chemotherapy (HR: 8.489, 95% CI: 1.637–44.016; *P* = 0.011), and qi deficiency (HR: 0.068, 95% CI: 0.010–0.460; *P* = 0.006) (Table 2).

**Table 2:** Prognostic risk factors for survival at 12 months in patients with lung cancer in the matched data (*n* = 54)

	Univariate			Multivariable		
Variables	HR	95% CI	*P*‐value	HR	95% CI	*P*‐value
Age (years)	–	–	–	–	–	–
> 65	1.484	0.484–4.552	0.490	0.287	0.042–1.976	0.205
Gender	–	–	–	–	–	–
Female	1.560	0.549–4.431	0.404	2.232	0.609–8.183	0.226
Preoperative BMI	–	–	–	–	–	–
≥ 22 (kg/m2)	1.410	0.520–3.822	0.499	0.755	0.208–2.734	0.668
Smoke	–	–	–	–	–	–
Yes	1.652	0.539–5.069	0.380	9.606	1.182–78.061	0.034
ILD	–	–	–	–	–	–
Yes	1.679	0.621–4.540	0.307	4.707	0.896–24.734	0.067
Pathological	–	–	–	–	–	–
SCLC	5.468	1.858–16.091	0.002	8.489	1.637–44.016	0.011
TNM stage	–	–	–	–	–	–
II	–	–	0.849	–	–	0.138
III	1.380	0.366–5.206	0.634	1.122	0.260–4.843	0.877
IV	1.076	0.269–4.304	0.918	0.206	0.028–1.524	0.122
Chemotherapy	–	–	–	–	–	–
No	0.462	0.163–1.311	0.147	0.151	0.027–0.851	0.032
Radiotherapy	–	–	–	–	–	–
No	0.887	0.328–2.400	0.814	0.534	0.121–2.359	0.408
Biotherapy	–	–	–	–	–	–
No	0.043	0.000–77.552	0.412	0.000	0.000	0.985
Surgery	–	–	–	–	–	–
No	0.284	0.038–2.143	0.222	0.288	0.029–2.884	0.289
CCD	–	–	–	–	–	–
Yes	1.146	0.442–2.972	0.779	4.570	0.926–22.554	0.062
Syndrome of TCM	–	–	–	–	–	–
Qi deficiency	0.249	0.057–1.091	0.065	0.068	0.010–0.460	0.006
RA	–	–	–	–	–	–
Yes	1.223	0.452–3.310	0.691	2.367	0.674–8.309	0.179

## Discussion

Lung cancer is the leading cause of cancer death worldwide, responsible for nearly 18% of all new cancer deaths.^6^ The current research on lung cancer mainly covers the areas of inflammation and immunity. Chronic inflammation leads to excessive tissue remodeling, loss of tissue architecture, and modifications to DNA and proteins as a consequence of oxidative stress, which all increase the risk for cancer development.^7^ Further, the imbalance between oncogenes and tumor‐suppressor genes is another area of interest. RA combined with lung cancer yields a special kind of lung cancer presentation that can adversely impact the life expectancy of lung cancer patients.^8^, ^9^ This phenomenon may occur through the destruction of the human body's immune system. ILD is a common intrapulmonary secondary disease in RA patients that not only reduces RA patients' survival time but may also increase the risk of patients suffering from cancer.^10^, ^11^ Such may be related to the patient's environment, occupational exposure, and diffuse inflammation.^12^

As can be seen from the results of our study, RA has an effect on the survival of patients with lung cancer, which is the same as seen in previous studies. RA is a chronic systemic inflammatory disease, and chronic inflammation can lead to the dysregulation of cytokines and chemokines.^13^ On one hand, the dysregulation of cytokines and chemokines can release inflammatory cells, such as macrophages and neutrophils, that are recruited and which produce many proinflammatory cytokines, such as interleukin (IL)‐1, IL‐6, and tumor necrosis factor alpha,^14^ and produce tumor‐promoting cytokines that activate transcription factors, such as nuclear factor kappa B (NF‐κB), STAT3, and activator protein‐1 (AP‐1),^15^ to promote tumor proliferation and metastasis. On the other, it has been shown to promote angiogenesis in vivo and increase the probability of tumor metastasis.^16^, ^17^

The excessive release of inflammatory factors leads to the breakdown of the body's immune system, which can act against citrullinated proteins' response in the lungs, leading to the occurrence of ILD.^18^ In our study, we can see that ILD has a greater impact on the survival time of RA patients with lung cancer than that in lung cancer patients without RA. The reason for this result is that RA‐ILD as a chronic proliferative disease has a poor prognosis; this further complicated with lung cancer means that the patient is in a particularly serious state. Previous studies have suggested that the core of the ILD and lung cancer copathogenesis is the irreversible loss of the capacity of the cell to grow and divide. ILD may lead to genetic and epigenetic alterations as well as the abnormal activation of common transduction pathways, including Wnt/b‐catenin and phosphoinositide 3‐kinase/protein kinase B.^19^ There are other scholars using microsatellite DNA analysis, whereby they have observed a loss of heterozygosity in MYCL1, FHIT, SPARC, p16Ink4, and TP53 genomic loci among ILD patients.^20^ Cell senescence and irreversible damage, genetic and epigenetic alterations, the activation of various information pathways, and an absence of a gene location will result in an imbalance between oncogenes and tumor‐suppressor genes, leading to tumorigenesis.

In our study, we introduced the concept of qi deficiency in TCM. We observed clinically that, with the progression of RA, patients will display clinical symptoms of qi deficiency. When the clinical symptoms of lung cancer patients appear to indicate qi deficiency manifestation, this suggests a serious situation of cardiopulmonary insufficiency. As can be seen from our results, when patients exhibit clinical symptoms of qi deficiency, their survival prognosis is worse than that of patients without qi deficiency. In this context, RA as an influencing factor is less important; the individual's cardiopulmonary insufficiency and serious health state play a more important role in their poor survival prognosis. The reason for this finding may be that when the lung function and heart function of the patient are damaged, the patient is in a state of immunosuppression or impaired immunity, which will lead to an imbalance between oncogenes and tumor‐suppressor genes in the patient, promoting the occurrence and metastasis of the tumor.^16^

To further validate our results, we performed a prognostic risk factor analysis for survival in lung cancer patients within one year. As can be seen from our results, SCLC was a risk factor for survival at the time of univariate analysis. SCLC is a unique form of lung cancer characterized by rapid growth, but with responsiveness to both radiotherapy and chemotherapy. However, if left untreated, SCLC results in the shortest survival of any pulmonary neoplasm.^21^ This is consistent with our results. Next, we conducted a multivariate analysis of all the factors, and the results revealed that smoking, the inability to receive chemotherapy, and qi deficiency were factors that affected the survival and prognosis of lung cancer patients. Cigarette smoke contains 4000 toxic substances, including nicotine, carcinogens, organic compounds, and free radicals.^22^ Chronic smoking can cause disorders in the release of inflammatory cytokines, which can lead to oxidative stress and may activate redox‐sensitive transcription factors, such as NF‐κB and activator protein‐1,^23^ as well as promote tumor development. Chemotherapy is a viable treatment for lung cancer that significantly increases the life expectancy of patients.^24^ Those who cannot receive chemotherapy, however, often present with a poor general status and cannot tolerate the side effects of chemotherapy. As such, the cancer cells are not well inhibited, leading to tumor metastasis, thus reducing the life cycle of these individuals. The observed qi deficiency results further verified our conclusion that qi deficiency has an impact on the survival of lung cancer patients.

Our study has several limitations that should be mentioned. First, this was a retrospective study, and although we used PSM to balance the baseline of each group, we could not completely exclude the influence of all confounders. Second, we analyzed data only for patients who had been seen in the Oncology Department of TJTCM in the previous three years. Third, the data of patients with ILD are few in number, which did not effectively reflect the impact of this disease on the survival of RA patients with lung cancer.

In conclusion, the survival prognosis of lung cancer patients with RA is worse than that in those without RA as a comorbidity. Further, ILD and qi deficiency are associated with reduced survival rates among patients with lung cancer and RA as opposed to without RA. In the context of copresenting lung cancer and RA, ILD and qi deficiency (cardiopulmonary insufficiency) take on more burden for the survival prognosis of these patients.

## Disclosure

No authors report any conflict of interest.
